# Association Between Morning Serum Cortisol and Burnout Syndrome in Healthcare Workers in Boyacá, Colombia: A Cross-Sectional Study

**DOI:** 10.3390/ijerph23080995

**Published:** 2026-07-29

**Authors:** Juan Pablo Carvajal, Fred Gustavo Manrique-Abril

**Affiliations:** 1 Carvajal Laboratorios IPS SAS, Tunja 150003, Boyacá, Colombia; 2 School of Business Administration, Faculty of Business Administration, Universidad de Manizales, Manizales 170004, Caldas, Colombia; 3 School of Medicine, Faculty of Health Sciences, Universidad Pedagógica y Tecnológica de Colombia, UPTC, Tunja 150003, Boyacá, Colombia; 4 Faculty of Nursing, Universidad Nacional de Colombia, Bogota 111321, Colombia

**Keywords:** occupational health, healthcare professional, burnout, job satisfaction, hydrocortisone, MBI-GS

## Abstract

**Highlights:**

**Public health relevance—How does this work relate to a public health issue?**

- Examines occupational burnout and physiological stress in healthcare workers, a critical but underrepresented group in public health research.
- Addresses mental health in the workplace within a Colombian context, contributing evidence from a low-represented region for such studies.

**Public health significance—Why is this work of significance to public health?**

- Provides empirical evidence of low burnout prevalence but identifies a meaningful subgroup with elevated emotional exhaustion, supporting early prevention approaches rather than reactive interventions.
- Contributes novel data from an underrepresented occupational sector and geographic region, strengthening the global evidence base on occupational mental health in low- and middle-income settings.

**Public health implications—What are the key implications or messages for practitioners, policy makers and/or researchers in public health?**

- Supports multilevel organizational strategies—workload management, job stability, role clarity, and psychosocial support—to protect worker mental health.
- Encourages future public health research to use longitudinal designs and multi-biomarker approaches when evaluating stress and burnout in occupational settings.

**Abstract:**

The present study evaluated the association between morning serum cortisol levels and burnout syndrome among healthcare workers in the department of Boyacá, Colombia. An observational, analytical, cross-sectional, and quantitative study was conducted on 69 employees. Total serum cortisol was measured via chemiluminescence in fasting morning samples (reference: 110–692 nmol/L). Burnout was assessed using the Maslach Burnout Inventory–General Survey (MBI-GS). A total of 96.6% of participants fell within the normal range for serum cortisol. MBI-GS dimensions reported mean scores of: exhaustion 1.54 ± 1.17, cynicism 0.93 ± 0.89, and professional efficacy 5.60 ± 0.60. High exhaustion prevalence was 16.9%, high cynicism 6.8%, and low efficacy 3.4%. Profile classification showed 67.8% “engaged,” 13.6% “overextended/worn-out,” and 5.1% with “burnout.” No significant association was found between cortisol and burnout dimensions: exhaustion (*r* = 0.069, *p* = 0.605), cynicism (r = −0.135, *p* = 0.309), and professional efficacy (*r* = 0.044, *p* = 0.742). Mean cortisol comparisons between high and low dimension groups showed no statistically significant differences (*p* > 0.05). The study documented a low prevalence of established burnout and an absence of significant association between morning serum cortisol and MBI-GS dimensions, suggesting a relatively healthy work environment; however, the prevalence of high emotional exhaustion warrants preventive interventions. Multilevel organizational strategies like workload management, job stability, role clarity, and psychosocial support are recommended for worker mental health protection.

## 1. Introduction

Burnout syndrome is an occupational phenomenon of increasing relevance in public health, characterized by emotional exhaustion, depersonalization or cynicism, and reduced professional efficacy [1,2]. Recognized by the World Health Organization in the International Classification of Diseases (ICD-11) as an occupational phenomenon resulting from chronic workplace stress that has not been successfully managed [2,3], burnout represents a significant risk factor for multiple adverse health outcomes, including cardiovascular disease, metabolic disorders, and impaired mental health [2,4]. Recent epidemiological evidence documents alarmingly high prevalence rates of burnout across diverse occupational settings. A systematic meta-analysis evaluating healthcare workers during the COVID-19 pandemic reported a pooled burnout prevalence of 52% (95% CI: 40–63%) [1]. In Latin America, a multicenter study involving 5437 healthcare professionals from six countries reported a burnout prevalence of 59.8%, with higher risk observed among public sector workers, women, and younger personnel [5,6,7]. In Colombia, reported prevalence rates range from 33% among surgical residents to as high as 98% among healthcare personnel, depending on the instruments used and the populations studied [8,9,10].The heterogeneity of cortisol findings in burnout (ranging from hypocortisolism to hyperactive patterns) suggests individual differences in hypothalamic–pituitary–adrenal (HPA) axis dysregulation.

The physiological response to chronic stress characteristic of burnout is mediated by sustained activation of the hypothalamic–pituitary–adrenal (HPA) axis [2,4]. The HPA axis follows a characteristic circadian rhythm, with peak cortisol concentrations occurring in the early morning hours (approximately 6:00–8:00 AM) and nadir levels around midnight [11,12,13]. Under conditions of chronic occupational stress, prolonged HPA axis activation may lead to dysregulation patterns, including a blunted cortisol awakening response, flattening of the diurnal slope, or sustained elevation of basal cortisol levels [4,11,14]. Serum cortisol is a well-established biomarker of HPA axis activity [15,16,17]. However, the relationship between cortisol levels and burnout has yielded heterogeneous results in the literature. A study conducted among Argentine healthcare workers found elevated hair cortisol levels in 10% of participants, with significant associations between cortisol concentration and emotional exhaustion [18]. Conversely, other studies have reported weak or null associations, suggesting that this relationship may be modulated by additional factors [15,16,17], such as individual adaptive capacity, chronotype, and specific characteristics of the occupational stressor [19,20,21].

The Maslach Burnout Inventory, in its General Survey version (MBI-GS), is the most widely used psychometric instrument for assessing burnout across diverse occupational populations [21,22]. In the Latin American context, validation studies have confirmed its applicability, although certain psychometric limitations have been reported, such as the low internal consistency of the depersonalization (cynicism) dimension, which often presents marginal Cronbach’s alpha values frequently situated around 0.60 in systematic reviews and as low as 0.40 in specific regional samples [21,23,24]. Furthermore, deficiencies in factorial validity due to cross-loadings of specific items have been identified, particularly for items 12 and 16, whose removal has been recommended to significantly improve model fit in Hispanic populations [20,21,22,23]. Contemporary evidence emphasizes the need for organizational-level interventions that address the structural and systemic factors contributing to burnout [25,26,27]. A meta-analysis of organizational interventions identified moderate but statistically significant effects, particularly when interventions targeted multiple organizational levels simultaneously. Specifically, effective organizational strategies include the optimization of clinical workflows through evidence-based process improvements, the integration of team-based care models—notably through the utilization of medical assistants or scribes to alleviate the clerical burden of electronic health records—and the implementation of interrupted staffing rotations to optimize workload distribution. Furthermore, institutional initiatives that enhance professional autonomy and provide protected time for peer interaction have demonstrated greater long-term efficacy in reducing emotional exhaustion and cynicism, emphasizing that systemic reorganization is significantly more effective than addressing burnout as a purely individual deficit [26,27,28].

Despite the growing body of evidence on burnout, a knowledge gap persists regarding workers in the healthcare sector, particularly in underrepresented regions for this kind of studies such as those in Colombian. The present study aimed to estimate the occurrence of burnout among 69 employees of a healthcare workers in the department of Boyacá, Colombia, between May and September 2025. We evaluated the association between morning serum cortisol levels and burnout syndrome as assessed using the MBI-GS. This study contributes to the recognition of occupational health conditions in laboratory settings, highlights the need for targeted interventions, and informs potential strategies to improve workplace well-being. This study was approved by the Institutional Review Board (IRB) of Institutional Review Board of Carvajal Laboratorios IPS SAS with approval number ICL-2025-001 dated 15 January 2025. All participants provided written informed consent.

## 2. Materials and Methods

### 2.1. Study Population

The present study was performed in Carvajal Laboratories IPS SAS, a private clinical laboratory company, with a workforce of 180 employees, as of 2025, and is headquartered in Tunja (population approximately 170.000), the capital city of the department of Boyacá, located in the Andean region of Colombia. This study was designed as an observational, analytical, cross-sectional, and quantitative study conducted between May and September 2025. The study population was determined through census sampling, including all workers who met the eligibility criteria during the study period. Given the ethical implications of collecting these data, the study protocol received prior approval from the institutional ethics committee and complied with the principles of the Declaration of Helsinki and Resolution 8430 of 1993 of the Colombian Ministry of Health, as well as Resolution 2646 of 2008 on psychosocial risk factors [29]. All participants signed informed consent forms before initiation of data collection and subsequent analyses and confidentiality was ensured through data anonymization. All participants completed all survey items. No missing data required imputation.

Inclusion criteria comprised: (i) being an active employee of Carvajal Laboratories IPS SAS during the study period; (ii) providing written informed consent; and (iii) availability to complete the Maslach Burnout Inventory–General Survey (MBI-GS) and to provide a blood sample for cortisol determination. Exclusion criteria included: absence during the data collection period; incomplete questionnaires; pregnancy or lactation; night-shift work or rotating shifts (due to potential circadian rhythm disruption); and a prior diagnosis of medical conditions known to affect the hypothalamic–pituitary–adrenal (HPA) axis, including Cushing’s syndrome, Addison’s disease, hypothyroidism, hyperthyroidism, and type 1 diabetes mellitus. Additionally, individuals using medications that may alter HPA axis function (systemic corticosteroids, oral contraceptives, hormone replacement therapy, antidepressants, anxiolytics, or antipsychotics), as well as those with major psychiatric disorders (major depressive disorder, bipolar disorder, or anxiety disorders under pharmacological treatment), were excluded.

### 2.2. Research Variables

Burnout was assessed using the Maslach Burnout Inventory–General Survey (MBI-GS), previously validated for Latin American populations [24]. The MBI-GS consists of 16 items evaluating three dimensions: (1) emotional exhaustion (5 items); (2) cynicism/depersonalization (5 items); and (3) professional efficacy (6 items). Each item is scored on a 7-point Likert scale ranging from 0 (“never”) to 6 (“every day”). Participants were classified as having a burnout profile when they simultaneously presented high emotional exhaustion (≥3.0), high cynicism (≥2.6), and low professional efficacy (≤4.0), in accordance with established cut-off values [21,23]. Internal consistency of the instrument in the study sample was assessed using Cronbach’s alpha [10,23].

Morning serum cortisol was measured as total cortisol concentration using a chemiluminescence immunoassay (functional sensitivity < 1 µg/dL). Participants were instructed to fast for at least 8 h prior to sampling, avoid vigorous physical exercise for 24 h, sleep a minimum of 6 h the night before, and refrain from caffeine, alcohol, and tobacco consumption for at least 12 h prior to blood collection. All participants rested for at least 15 min before venipuncture. Blood samples were obtained in the fasting state during the morning period (8:00–9:00 AM) to capture the circadian cortisol peak, via antecubital venipuncture. Serum was separated by centrifugation at 3000 rpm for 10 min within 2 h of collection and stored at −20 °C until analysis by chemiluminescence. Morning reference values of 110–692 nmol/L (5–25 µg/dL) were used [15,17]. Cortisol levels were categorized as low (<110 nmol/L), normal (110–692 nmol/L), or high (>692 nmol/L).

Sociodemographic, occupational, and lifestyle data were collected through a structured digital questionnaire. The instrument comprised seven sections: (1) Identification data, (2) Sociodemographic data (age, sex, marital status, educational level, profession, number of economic dependents, household head status, number of cohabitants, and housing type), (3) Occupational information (position, department, tenure at current company and total work experience, contract type, work shift, weekly work hours, overtime work, commute time, moonlighting, salary satisfaction, job autonomy, perceived workload, supervisor and peer support, and workplace conflicts), (4) Lifestyle habits (sleep hours, sleep quality, current studies, physical activity, recreational activities, alcohol and tobacco consumption, and domestic task support), (5) Health and wellbeing (chronic diseases, medication use, body mass index, medical leave, mental health care, and perceived health status), (6) Psychosocial factors and stress (social support network, turnover intention, perceived stress level, stressful life events, and work–life balance), and (7) Clinical data (serum cortisol, sampling time, and MBI-GS scores). Responses were coded according to a predefined variable dictionary and stored digitally for subsequent statistical analysis.

### 2.3. Statistical Analysis

Descriptive statistics were used to characterize the study population. Categorical variables were summarized using absolute and relative frequencies, whereas continuous variables were described using means and standard deviations (SD). The distribution of continuous variables was assessed for normality using the Kolmogorov–Smirnov test, and homogeneity of variances was evaluated using Levene’s test. When normality and homoscedasticity assumptions were met, parametric tests were applied; otherwise, non-parametric alternatives were used. Associations between morning serum cortisol levels and burnout dimensions (emotional exhaustion, cynicism, and professional efficacy) were evaluated using Pearson’s correlation coefficient for normally distributed variables and Spearman’s rank correlation coefficient when normality assumptions were violated. Correlation coefficients were reported together with their corresponding *p* values.

Comparisons of mean cortisol levels between groups, classified as having high versus low scores in each burnout dimension, were performed using Student’s *t* test for independent samples. When assumptions for parametric testing were not satisfied, the Mann–Whitney U test was applied. Mean differences were reported with 95% confidence intervals (95% CI). Effect sizes for group comparisons were calculated using Cohen’s d to estimate the magnitude of differences in cortisol levels between burnout categories. Statistical significance was defined as a two-tailed *p* value < 0.05, with the significance level set at α = 0.05. All statistical analyses were conducted using Python (version 3.12).

## 3. Results

The final sample from Carvajal Laboratories IPS SAS (Table 1) comprised 69 healthcare workers with a mean age of 36.0 ± 7.5 years (range: 22–56 years) and with a majority of women (*n* = 53, 76.8%). Regarding marital status, 44.9% (*n* = 31) were single, 31.9% (*n* = 22) married or in a domestic partnership, 18.8% (*n* = 13) widowed, and 4.3% (*n* = 3) separated or divorced. The predominant educational level was technical/technological (43.5%, *n* = 30), followed by specialization (24.6%, *n* = 17), undergraduate degree (18.8%, *n* = 13), master’s degree (7.2%, *n* = 5), and secondary education (5.8%, *n* = 4). Participants reported a mean of 1.5 ± 1.1 economic dependents, and 40.6% (*n* = 28) identified themselves as household heads. Concerning occupational characteristics, the mean tenure at the current organization was 49.3 ± 40.7 months (approximately 4.1 years), with total work experience of 7.7 ± 6.7 years. The majority held permanent contracts (91.3%, *n* = 63) with day shifts (88.4%, *n* = 61). Mean weekly working hours were 40.6 ± 11.3 h, and 46.4% (*n* = 32) reported working overtime. Only 2.9% (*n* = 2) held an additional job. Regarding health and lifestyle, participants slept an average of 6.1 ± 1.1 h per night and engaged in 1.2 ± 2.2 h of physical activity per week. Mean body mass index was 24.0 ± 5.4 kg/m^2^, and 11.6% (*n* = 9) reported having at least one chronic disease. For work-related psychosocial variables, mean scores were: salary satisfaction 3.4 ± 0.9 (on a 1–5 scale), perceived workload 3.5 ± 0.8, supervisor support 3.7 ± 1.0, perceived stress level 2.9 ± 1.0, and work–life balance 3.4 ± 1.0. Additionally, 18.8% (*n* = 13) had considered resigning in the past six months.

The mean morning serum cortisol concentration was 222.77 ± 102.45 nmol/L (median: 198.00 nmol/L; range: 70.00–566.00 nmol/L). Based on established morning reference values (110–692 nmol/L), 96.6% (*n* = 57) of participants presented cortisol levels within the normal range, while 3.4% (*n* = 2) exhibited low cortisol levels. No cases of elevated cortisol were identified (Table 1). Mean scores for the MBI-GS dimensions were 1.54 ± 1.18 for emotional exhaustion, 0.93 ± 0.89 for cynicism, and 5.60 ± 0.60 for professional efficacy (Table 1). According to established cut-off values, 17.9% (*n* = 11) of participants exhibited high emotional exhaustion (≥3.0), 6.8% (*n* = 4) high cynicism (≥2.6), and 3.4% (*n* = 2) low professional efficacy (≤4.0). Classification based on MBI-GS profiles showed that 67.8% (*n* = 40) were classified as engaged, 13.6% (*n* = 9) as overextended, 10.2% (*n* = 6) as disengaged, 5.1% (*n* = 3) as burnout, and 3.4% (*n* = 2) as ineffective (Table 1). When applying the strict burnout criteria—simultaneous presence of high emotional exhaustion, high cynicism, and low professional efficacy—no participant (0%) met all three conditions concurrently. Pearson correlation analyses between serum cortisol levels and burnout dimensions showed no statistically significant associations: emotional exhaustion (*r* = 0.069, *p* = 0.605), cynicism (*r* = −0.135, *p* = 0.309), and professional efficacy (*r* = 0.044, *p* = 0.742). Spearman rank correlation analyses yielded similarly non-significant results (Table 2). Detailed descriptive statistics and effect sizes by MBI-GS profile are provided in Supplementary Materials (Tables S1 and S2).

Comparisons of cortisol levels between participants with high versus low scores in each burnout dimension revealed no statistically significant differences. Mean cortisol levels among participants with high emotional exhaustion were 199.46 ± 70.55 nmol/L, compared with 227.53 ± 107.76 nmol/L among those with low emotional exhaustion (*p* = 0.435). For cynicism, mean cortisol levels were 164.15 ± 60.87 nmol/L in the high cynicism group and 227.04 ± 103.89 nmol/L in the low cynicism group (*p* = 0.239). Participants with low professional efficacy had mean cortisol levels of 211.50 ± 88.39 nmol/L, compared with 223.17 ± 103.57 nmol/L among those with high professional efficacy (*p* = 0.876) (Table 3).

**Figure 1:**
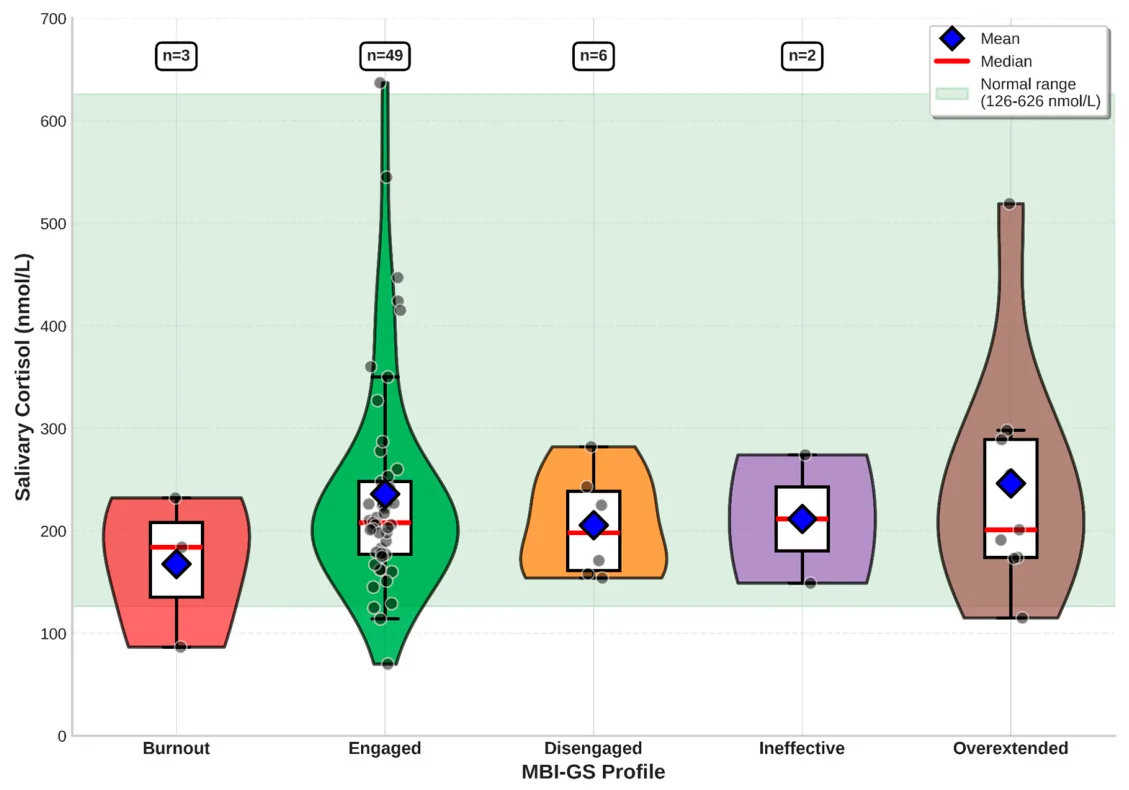
Distribution of morning serum cortisol levels across MBI-GS profiles (*N* = 69). Violin plots represent the distribution of cortisol values within each profile. Boxes indicate the interquartile range (IQR), with the median shown as a red line; blue diamonds represent the mean, and individual data points are displayed. The shaded green area indicates the reference range for serum cortisol (126–626 nmol/L). Sample sizes are provided for each group. Colors represent different MBI-GS profile categories (Engaged, Overextended, Disengaged, Burnout, and Ineffective). No statistically significant differences were observed between groups (ANOVA: F(4,64) = 0.46, *p* = 0.764; Kruskal–Wallis: H(4) = 1.23, *p* = 0.873).

Figure 1 further illustrates the distribution of morning serum cortisol levels across MBI-GS profiles (*n* = 69). Cortisol values showed substantial overlap between groups, with most observations falling within the reference range (126–626 nmol/L). No statistically significant differences were observed between profiles (one-way ANOVA: F(4,64) = 0.46, *p* = 0.764, η^2^ = 0.028), a finding that was consistent with the Kruskal–Wallis test (H(4) = 1.23, *p* = 0.873). Comparisons between the burnout and engaged profiles also revealed no significant differences (Welch’s t = −1.51, *p* = 0.245), despite a moderate effect size (Cohen’s d = −0.74).

## 4. Discussion

This study represents one of the first evaluations of the association between serum cortisol and burnout among healthcare workers in Colombia, conducted using an internationally validated instrument (MBI-GS), objective cortisol measurement, and census sampling in an occupational sector and geographic region underrepresented in the literature. Our findings indicate a low prevalence of burnout according to strict MBI-GS criteria (0.0–5.1%) and no statistically significant associations between morning serum cortisol levels and burnout dimensions or profiles. As shown in both the group comparisons (Table 3) and the distribution analysis (Figure 1), cortisol values exhibited substantial overlap across all burnout categories, with most observations remaining within the physiological reference range. These results suggest the absence of marked HPA axis dysregulation at the group level within this population. Although the cross-sectional design precludes causal inference, the overall pattern is consistent with a work environment predominantly characterized by favorable occupational profiles, with 67.8% of workers classified as engaged. Nevertheless, the identification of elevated emotional exhaustion in 16.9% of participants underscores the need for preventive strategies. Post hoc power analysis revealed 18% power, well below the 80% threshold. Given the small sample size (*n* = 69) and low burnout prevalence (*n* = 3), non-significant findings should be interpreted cautiously as exploratory results requiring confirmation in larger studies.

The prevalence of burnout observed in this study (0.0% using strict criteria; 5.1% based on MBI-GS profiles) contrasts sharply with reports from Latin American healthcare workers, where prevalence estimates range from 39.8% to 59.8% [5,6], and with Colombian studies reporting values between 33% and 98% [8,9,10]. Several factors may account for this substantial discrepancy. First, the occupational context of industrial clinical laboratory personnel differs from that of frontline healthcare providers, as it generally involves lower direct emotional demands, reduced exposure to critical patient care, and more structured task-oriented workflows [1,5,6,24]. Second, organizational characteristics of Carvajal Laboratories IPS SAS may reflect a work environment with effective resource management, role clarity, and organizational support. These elements function as critical protective buffers by maintaining a sustainable equilibrium between workload demands and available professional resources [27]. Specifically, transformational leadership and participatory management facilitate a healthy organizational culture, while evidence-based workflow optimizations and team-based collaboration effectively minimize the clerical and emotional burdens that often precipitate burnout in clinical settings [25,28]. Such structural factors foster a sense of community and value alignment, which are essential for sustaining high levels of work engagement and preventing the systemic progression of occupational stress [27]. Third, the high proportion of permanent employment contracts (91.5%) suggests greater job stability, which has been associated with reduced burnout risk [19,24]. Despite these favorable conditions, the proportion of workers experiencing high emotional exhaustion (16.9%) and those classified as overextended (13.6%) indicates that a meaningful subset of employees may be at risk of progression toward established burnout if preventive measures are not implemented.

The absence of statistically significant associations between morning serum cortisol levels and burnout dimensions (Figure 1) is consistent with some prior studies [4,11], although it contrasts with others reporting positive associations [2,16]. This heterogeneity likely reflects both methodological and the complex physiology of the hypothalamic–pituitary–adrenal (HPA) axis. From a pathophysiological perspective, the relationship between cortisol and burnout is complex and not necessarily linear. The literature describes distinct phases of hypothalamic–pituitary–adrenal (HPA) axis response, beginning with an initial hyperactivation phase characterized by elevated cortisol, followed by potential hypoactivation and reduced cortisol levels in cases of severe and prolonged stress. A meta-analysis reported only a weak association between morning cortisol and HPA axis activity, suggesting limited sensitivity of single-point measurements [11,12].

In the present study, the low prevalence of burnout may partly explain the lack of association. Moreover, although morning serum cortisol measurement is appropriate for capturing the circadian peak, it represents a single time point within a highly dynamic system. Future studies in this regional context should consider multiple daily measurements (morning, afternoon, and evening) or the use of chronic exposure biomarkers, such as hair cortisol, which reflects cumulative cortisol secretion over several months [15,20,30]. Additionally, circadian rhythm dysregulation—manifested as a flattened diurnal cortisol slope—may be a more sensitive indicator of chronic stress than absolute morning cortisol levels [11,12,16]. Individual-level factors, including chronotype, sleep quality, body mass index, and menstrual cycle phase, may also influence cortisol concentrations independently of occupational stress [11,13,14]. The high proportion of female participants (78.0%) may therefore introduce additional biological variability that warrants consideration in future research. Additionally, a closer examination of individual profiles with the most conspicuous values (outliers) reveals specific contextual factors that account for the data dispersion observed in Figure 1. These included personal health challenges, specifically cases involving chronic illness and long-term treatment with a high emotional burden. Such factors have been identified as significant biological confounders in the assessment of the HPA axis, as they can influence cortisol concentrations independently of occupational stress [11]. Additionally, job-specific stressors observed in leadership roles—such as role ambiguity where technical staff assume professional-level responsibilities, and a lack of control over external variables like specialist agendas—likely contributed to elevated exhaustion scores in specific cases [27]. Furthermore, instances of temporary peaks in organizational responsibility without adequate personnel support illustrate how structural imbalances precipitate strain, while also highlighting the importance of proactive workload adjustments, such as subsequent staff expansion [25]. These cases underscore the necessity of considering individual life-histories and the specific dynamics of work units when interpreting neuroendocrine markers [12].

These findings have several implications for occupational health management in Colombia. First, the low prevalence of burnout and high proportion of engaged workers suggest a relatively healthy work environment that could serve as a reference model for other organizations. Second, the identification of a subgroup with elevated emotional exhaustion highlights the importance of early preventive interventions to avoid progression toward full burnout. Third, the lack of association between cortisol and burnout suggests that alternative biomarkers or complementary clinical and psychosocial indicators may be more informative for monitoring occupational mental health. Although several major confounders were addressed through exclusion criteria, other relevant variables—such as sleep quality, chronotype, body mass index, menstrual cycle phase, and use of non-prescription medications may have influenced cortisol levels independently of occupational stress. Despite these limitations, the study provides novel evidence from an underrepresented sector and region, and offers a foundation for future longitudinal and multi-biomarker research in occupational mental health.

### Organizational Recommendations

Based on contemporary scientific evidence and the Colombian occupational context, the following recommendations are proposed:

1. Proactive workload management: Implement regular audits comparing workload demands with available capacity and adjust task allocation proactively when overload is detected.
2. Expansion of job autonomy and control: Increase employee control over work processes and schedules through autonomy-enhancing practices and self-management structures.
3. Consistent recognition systems: Implement formal recognition programs with explicit and equitable criteria, as insufficient recognition has been associated with a 45–48% increase in burnout risk [31].
4. Strengthening workplace relationships: Promote high-quality interpersonal relationships by fostering collaborative structures and psychological safety.
5. Role clarity: Establish clear job descriptions with defined responsibilities, authority, and performance metrics, given the strong association between role ambiguity and burnout.
6. Transformational leadership: Train supervisors to recognize early signs of burnout, engage in supportive conversations about mental health, and provide emotional support.
7. Digital boundary management: Establish clear policies regarding after-hours communication and the right to disconnect.
8. Comprehensive well-being programs: Complement organizational interventions with periodic screening, access to psychological support services, and education on sleep hygiene.

Mindfulness-based interventions have demonstrated effectiveness in burnout prevention. A meta-analysis of 91 randomized controlled trials involving 4927 participants showed significant improvements in mindfulness, well-being, and mental health, along with substantial reductions in perceived stress [32,33]. In Colombia, a randomized controlled study evaluating a mindfulness-based health promotion program designed for Hispanic/Latin American populations reported significant benefits among hospital workers, including marked reductions in psychological distress and perceived stress [34]. In light of this evidence and the Colombian experience with this program, the following actions are recommended:

1. Implementation of a mindfulness-based health promotion program: Tailored to Hispanic/Latin American populations and validated in Colombian workers.
2. Program structure: An 8-week intervention with weekly sessions lasting 60–90 min, incorporating mindfulness practices, mindful movement, conscious eating, and psychoeducation.
3. Organizational integration: Delivery during working hours with protected time, explicit leadership support, and infrastructure for continued practice following program completion.
4. Systematic evaluation: Rigorous pre- and post-intervention assessment using validated instruments, with follow-up evaluations at 3 and 6 months.

## 5. Conclusions

This study documented a low prevalence of burnout syndrome (0.0–5.1% according to MBI-GS criteria) among clinical laboratory workers in Boyacá, Colombia, with a predominance of engaged occupational profiles (67.8%). Nevertheless, 16.9% of participants exhibited elevated emotional exhaustion, highlighting the need for ongoing surveillance and early preventive strategies. No statistically significant association was identified between morning serum cortisol levels and burnout, a finding that underscores the complexity of the relationship between biological stress markers and clinical manifestations of occupational stress.

Overall, the results suggest a relatively healthy work environment, while emphasizing the importance of targeted interventions for workers experiencing emotional exhaustion. Evidence-based recommendations include multilevel organizational interventions—such as workload management, enhanced autonomy, recognition, social support, and role clarity—combined with culturally adapted mindfulness-based programs, including the Mindfulness-Based Health Promotion program validated in Colombian populations. Future research should employ longitudinal designs, comprehensive assessment of HPA axis function, inclusion of additional stress biomarkers, psychometric validation of the MBI-GS for Colombian laboratory workers, and multilevel analytical approaches to advance the understanding and prevention of burnout in Colombian occupational settings.

## Figures and Tables

**Table 1:** Baseline characteristics of the study population (n = 69).

Characteristic	Value
Sex, *n* (%)	
Female	46 (78.0)
Male	13 (22.0)
Type of contract, n (%)	
Permanent contract	54 (91.5)
Fixed-term/task-based	5 (8.5)
*Job tenure (months)*	
Mean ± SD	38.9 ± 27.7
Median (IQR)	39.0 (15.5–51.5)
Morning serum cortisol (nmol/L)	
Mean ± SD	222.77 ± 102.45
Median (IQR)	198.00 (164.00–242.50)
Range	70.00–566.00
Cortisol category, n (%)	
Low (<110)	2 (3.4)
Normal (110–692)	57 (96.6)
High (>692)	0 (0.0)
MBI-GS dimensions, mean ± SD	
Emotional exhaustion (0–6)	1.54 ± 1.18
Cynicism (0–6)	0.93 ± 0.89
Professional efficacy (0–6)	5.60 ± 0.60
Prevalence of elevated dimensions, n (%)	
High emotional exhaustion (≥3.0)	10 (16.9)
High cynicism (≥2.6)	4 (6.8)
Low professional efficacy (≤4.0)	2 (3.4)
MBI-GS profile, *n* (%)	
Engaged	40 (67.8)
Overextended	9 (13.6)
Disengaged	6 (10.2)
Burnout	3 (5.1)
Ineffective	2 (3.4)

SD: standard deviation; IQR: interquartile range; MBI-GS: Maslach Burnout Inventory–General Survey; n: sample size. Italic text denotes variables measured on continuous scales for which the Mann–Whitney U test was applied in subsequent analyses.

**Table 2:** Pearson and Spearman correlation between serum cortisol and burnout dimensions (*n* = 610).

MBI-GS Dimension	Pearson		Spearman	
	Correlation (*r*)	*p*-Value	Correlation (ρ)	*p*-Value
Emotional exhaustion	0.069	0.605	0.227	0.084
Cynicism	−0.135	0.309	0.127	0.339
Professional efficacy	0.044	0.742	0.078	0.558

MBI-GS: Maslach Burnout Inventory–General Survey. No correlations reached statistical significance (*p* < 0.05).

**Table 3:** Morning serum cortisol levels according to burnout dimension categories (*n* = 69).

Variable	*n* (%)	Cortisol (nmol/L) Mean ± SD	*p*-Value
Emotional exhaustion			0.435
Low (<3.0)	49 (83.1)	227.53 ± 107.76	
High (≥3.0)	10 (16.9)	199.46 ± 70.55	
*Cynicism*			0.239
Low (<2.6)	55 (93.2)	227.04 ± 103.89	
High (≥2.6)	4 (6.8)	164.15 ± 60.87	
Professional efficacy			0.876
Low (≤4.0)	2 (3.4)	211.50 ± 88.39	
High (>4.0)	57 (96.6)	223.17 ± 103.57	

SD: standard deviation; *n*: sample size. Italic row labels indicate variables for which the Mann–Whitney U test was applied. *p*-values were obtained using Student’s *t* test for independent samples. No comparisons reached statistical significance (*p* < 0.05).

## Data Availability

The data sets generated and analyzed during the current study are not publicly available due to privacy/ethical restrictions.

## Supplementary Materials

The following supporting information can be downloaded at https://www.mdpi.com/article/10.3390/ijerph23080995/s1, Table S1. Descriptive Statistics by MBI-GS Profile, Table S2. Comparison Statistics and Effect Sizes.
