# Supplementary material for: Association Between Morning Serum Cortisol and Burnout Syndrome in Healthcare Workers in Boyacá, Colombia: A Cross-Sectional Study
Source: Int J Environ Res Public Health. 2026 Jul 29;23(8):995. doi: 10.3390/ijerph23080995 (PMC13512623; doi:10.3390/ijerph23080995)
Supplement: Supplementary file 1 [file ijerph-23-00995-s001.zip › ijerph-4334795-supplementary.pdf]

Table S1. Descriptive Statistics by MBI-GS Profile

MBI-GS Profile	N	Cortisol (nmol/L), Mean±SD	Emotional Exhaustion	Cynicism
Burned Out	3	4621.9±2043.9	3.47±1.03	3.07±0.31
Engaged	49	6506.8±2945.3	1.16±0.83	0.56±0.52
Disengaged	6	5669.3±1446.1	1.40±0.84	2.10±0.58
Overextended	9	6789.7±3262.6	3.40±0.62	1.44±0.93
Ineffective	2	5834.9±2438.5	0.60±0.57	0.90±0.99

Table S2. Comparison Statistics and Effect Sizes

Comparison	Test Statistic	p-value	Effect Size
Burnout vs Engaged (cortisol)	t=-1.10	p=0.276	d=-0.74
ANOVA (cortisol by profile)	F=0.46	p=0.764	η^2=0.028
Kruskal-Wallis (non-parametric)	H=1.23	p=0.873	-
Post-hoc Power Analysis	Power=18%	<0.80 target	Sample too small

Note: 95% Confidence Intervals, Cronbach's alpha, and adjusted regression models are included in the main manuscript revision.
